# Integrated Anthropometric, Physiological and Biological Assessment of Elite Youth Football Players Using Machine Learning

**DOI:** 10.3390/diagnostics15243238

**Published:** 2025-12-18

**Authors:** Luiza Camelia Nechita, Tudor Vladimir Gurau, Carmina Liana Musat, Ancuța Elena Țupu, Gabriela Gurau, Doina Carina Voinescu, Aurel Nechita

**Affiliations:** Faculty of Medicine and Pharmacy, ‘Dunarea de Jos’ University of Galati, 800008 Galati, Romania

**Keywords:** elite youth football, anthropometry, physical performance, biological markers, injury prevention, risk management

## Abstract

**Background:** Youth football players experience rapid physical and biological changes while being exposed to high training loads, increasing performance demands and musculoskeletal injury risk. Current evaluations often analyze anthropometric, physiological, and biological domains separately, and few studies integrate these dimensions using machine-learning (ML) approaches. **Objective:** To provide a multidimensional assessment of elite youth football players and investigate how anthropometric, physical, and biological markers jointly relate to performance through classical statistics and ML. **Methods:** One hundred elite players (14–18 years) underwent standardized anthropometric, physical, and laboratory assessments. Analyses included descriptive statistics, ANOVA/MANOVA, PCA, factor analysis, composite biological indices, and ML models (linear regression, SVR) with 5-fold cross-validation. K-means clustering explored hidden adaptation phenotypes. **Results:** Older players showed higher weight and BMI, physical testing revealed consistent limb asymmetry (~5%), and biological markers remained within reference ranges. PCA and factor analysis extracted latent structural and metabolic domains. Linear regression predicted performance with R^2^ ≈ 0.59, while SVR underperformed. K-means identified three adaptation phenotypes. **Conclusions:** Performance and resilience arise from interactions between structural, functional, and biological domains. Interpretable ML methods enhance individualized monitoring, early risk detection, and evidence-based injury-prevention strategies.

## 1. Introduction

Football is the most practiced sport worldwide, with over 250 million registered players and millions of children and adolescents engaged in structured training programs [1]. Elite youth football players represent a highly specific category, characterized by intense training regimens, early specialization, and exposure to competitive demands during critical phases of growth and biological development [2,3]. At this stage, anthropometric, physiological, and biological factors undergo rapid changes, influencing both athletic performance and susceptibility to musculoskeletal injuries.

In elite academies, systematic evaluation of young players is crucial not only for optimizing performance but also for preventing injuries and managing long-term health risks [4]. Musculoskeletal injuries represent one of the leading causes of absenteeism in youth football, with significant consequences for individual careers and for the overall efficiency of talent development programs. Recent reports indicate that overuse injuries, asymmetries in muscular strength, and imbalances between growth-related body proportions are major determinants of injury risk in adolescent athletes [5].

Traditionally, player assessment has been conducted within three separate domains: (i) anthropometric—focusing on growth, proportionality, and body composition; (ii) physiological—monitoring muscular strength, flexibility, and neuromuscular performance [6]; and (iii) biological—tracking hematological, biochemical, and inflammatory markers [7,8,9]. While each domain provides valuable insights, isolated evaluation limits the understanding of complex interactions across structural, functional, and metabolic systems. Moreover, traditional statistical methods, although robust, often lack the capacity to capture multidimensional interactions and hidden patterns within integrated datasets.

In recent years, artificial intelligence (AI) and machine learning (ML) have emerged as powerful tools for medical and sports sciences [10]. These methods enable dimensionality reduction, clustering of hidden subgroups, and predictive modeling of outcomes based on heterogeneous data [11]. Applications in football have mainly focused on performance analytics, GPS tracking, and injury surveillance, yet few studies have attempted to integrate anthropometric, physiological, and biological data into unified predictive frameworks [12]. Such integrative approaches hold potential for personalized monitoring, early detection of maladaptation, and evidence-based prevention strategies.

Recent interdisciplinary research has also shown that combining physiological, anthropometric, and metabolic markers through ML pipelines can substantially improve prediction of adaptation and injury risk. Studies such as [13,14] demonstrated that ML models incorporating multivariate structural and biochemical inputs outperform traditional assessments in identifying at-risk athletes and maturation-dependent performance profiles. Despite these advancements, existing approaches typically integrate only two domains at a time and rarely apply full-spectrum ML analysis including PCA, factor analysis, supervised modeling, and clustering.

Although previous studies have examined anthropometric development, neuromuscular capacity, and isolated biochemical markers, very few have combined all three domains into a single analytical framework while also leveraging AI-driven approaches to extract latent patterns and improve predictive accuracy. Most available studies rely exclusively on classical statistics and therefore cannot capture the multidimensional interactions that characterize adolescent athletes’ development. The present study addresses these gaps by integrating structural, functional, and biological data and applying PCA, factor analysis, k-means clustering, and supervised ML to elucidate latent domains and build interpretable performance models.

The gap in the literature lies precisely in the lack of multidisciplinary analyses that combine structural, functional, and metabolic information in adolescent athletes while simultaneously applying modern AI-driven approaches to improve interpretability and prediction accuracy [13,14]. Addressing this gap is essential, given that adolescence represents a critical window for talent development, where inadequate monitoring or missed warning signals can translate into long-term injury risk and underperformance.

The present study aims to fill this gap by providing a comprehensive, multidimensional evaluation of elite youth football players, combining anthropometric, physiological, and biological assessments under standardized protocols. Furthermore, advanced statistical and ML methods were employed to: (i) characterize group differences, (ii) identify hidden adaptation profiles, (iii) extract latent domains of variability, and (iv) build predictive and interpretable models of physical performance and injury-related indicators.

Through this integrative approach, the study advances the current understanding of how structural, functional, and biological factors interact in shaping performance outcomes and musculoskeletal resilience in adolescent football players. Ultimately, the results are expected to support medical staff, coaches, and sports scientists in designing evidence-based monitoring systems that leverage AI-enhanced tools for risk management and long-term injury prevention.

## 2. Materials and Methods

### 2.1. Dataset

The study included a cohort of 100 elite youth football players, aged 14–18 years, recruited from professional academies. Data collection was carried out according to the standardized CMS training and evaluation protocol. All measurements were performed in the morning, under fasting conditions, and after a minimum of 48 h from the last official match, in order to minimize the influence of acute fatigue and ensure standardized baseline values.

Inclusion criteria were: male players aged 14–18 years, active in elite academies, with ≥3 years of structured training, and no acute injury in the previous three months. Exclusion criteria included chronic illness, medication affecting metabolism or neuromuscular function, incomplete testing, or refusal to provide informed consent. No drop-outs occurred; all 100 participants completed the full testing protocol.

The dataset integrated three major domains of evaluation:

1. First, anthropometric parameters were assessed, including longitudinal, transversal, and sagittal body dimensions, as well as body weight and the Body Mass Index (BMI). These measurements were conducted following ISAK (International Society for the Advancement of Kinanthropometry) recommendations and complementary Romanian reference protocols. They provided information on proportional growth, musculoskeletal loading, and potential risk factors for postural imbalance or injury.
2. Second, physical parameters were evaluated through functional tests of muscular strength and spinal mobility. Specifically, handgrip strength (right and left) was measured with a digital dynamometer, lumbar strength with a back dynamometer, and spinal flexibility with a standardized flexometer. These parameters are critical in football, where repeated high-intensity actions (sprints, jumps, tackles) expose the musculoskeletal system to substantial stress. All physical tests were performed in duplicate, and the best value was retained for analysis. Reliability for handgrip and lumbar strength testing in adolescent athletes is high (ICC 0.86–0.94), and the flexometer method has established field validity, strengthening the reproducibility of the measurements.
3. Third, biological parameters were determined through hematological, biochemical, and urinary analyses, performed in an accredited clinical laboratory. The hematological profile included hemoglobin (Hb), hematocrit (Ht), and white blood cell count (WBC), reflecting oxygen transport capacity and immune status. The biochemical panel covered markers of metabolic control, liver and renal adaptation, and electrolyte balance, namely alanine aminotransferase (ALT/TGP), fasting glucose, high-density lipoprotein cholesterol (HDL), C-reactive protein (CRP), urea, creatinine (Cr), calcium (Ca), and magnesium (Mg). Finally, urinary markers were assessed, including urine specific gravity (USG) for hydration balance, proteinuria (ProtU) for renal/muscle integrity, and hematuria (HemU) as a potential indicator of microtrauma or dehydration.

All subjects and their parents or legal guardians provided written informed consent prior to participation. The study protocol was reviewed and approved by the Ethics Committee of the “Dunărea de Jos” University of Galați (approval codes: CEU Decision No. 31/20 June 2025 and CEU Decision No. 32/20 June 2025) and was conducted in accordance with the principles outlined in the Declaration of Helsinki.

Table 1 presents descriptive statistics (means, SDs, min–max ranges) for all anthropometric, physical, and biological variables included in the dataset.

### 2.2. Anthropometric Evaluation

Anthropometric assessment represents a cornerstone in evaluating the physical development and proportionality of elite youth football players [15]. Accurate measurement of body dimensions provides essential insights into growth, posture, musculoskeletal loading, and potential risk factors for injury [16]. All measurements were performed in the morning, barefoot, with subjects in standardized anatomical position, following internationally accepted anthropometric protocols.

#### 2.2.1. Physical Development Assessment

The evaluation of physical development included longitudinal, transversal, and sagittal body dimensions, each selected for their relevance to postural balance, musculoskeletal loading, and proportionality between body segments:

• Height (stature) was measured with a stadiometer, subjects standing barefoot with their heads in the Frankfurt plane. As an indicator of longitudinal growth, stature directly influences balance and the loading of lower limb joints, with extreme values predisposing athletes to increased stress on the knees and ankles.
• Arm span was determined using a rigid anthropometric rod, with arms fully extended laterally. This parameter serves as a marker of proportionality relative to height, where discrepancies may signal postural adaptations or shoulder asymmetry.
• Biacromial diameter (shoulder breadth) was assessed with a large caliper placed between the acromial landmarks. This measurement reflects scapular stability, and imbalances with pelvic width may predispose players to disharmony between the upper and lower girdles.
• Bitrochanteric diameter (Pelvic breadth) was measured between the trochanteric landmarks. As a determinant of hip–knee alignment, a wide pelvis may modify the Q-angle, thus increasing the risk of knee injuries.
• Thoracic diameters were assessed both transversally (mid-axillary line) and sagittally (between the xiphoid appendix and thoracic vertebrae), in both inspiration and expiration. These chest dimensions are closely linked to respiratory efficiency and posture; a narrow or flattened chest can reduce ventilatory capacity and promote postural imbalance.

This comprehensive anthropometric profiling allowed for a robust evaluation of proportionality and potential musculoskeletal risk factors in elite youth players. Normal reference values for anthropometric evaluation are provided in Table 2.

#### 2.2.2. Nutritional Status Assessment

Beyond skeletal proportionality, nutritional status was also assessed, as it provides complementary insights into energetic balance and muscular development [17]. Two main indicators were considered: body weight and the BMI, described in Table 3. These are widely used in sports science to evaluate the balance between body composition, muscular development, and energy availability [18]. Although BMI has limitations in athletic populations due to higher lean mass [19], it remains a useful screening tool when interpreted alongside performance-specific parameters (Table 4).

A correct evaluation of nutritional status thus complements anthropometric measurements, ensuring that both structural development and energetic balance are aligned with the physical demands of elite football.

### 2.3. Physical Evaluation

The physical evaluation focused on the functional assessment of the musculoskeletal system, with emphasis on muscular strength and spinal flexibility [20]. These parameters are essential for football players, where repetitive high-intensity movements (sprints, jumps, tackles) expose the body to substantial biomechanical stress. All evaluations were performed in standardized laboratory conditions, using validated protocols and calibrated instruments, in line with international recommendations [21,22]. Test–retest reliability values and methodological validity are well established for these tests in adolescent athletes, supporting methodological robustness.

#### 2.3.1. Handgrip Strength (Right/Left)

Handgrip strength was measured as an indicator of overall muscular fitness and neuromuscular function. Assessments were performed with a Grip D digital hand dynamometer (5–100 kg, accuracy 0.1 kg). Each player completed two trials per hand, with the highest value recorded.

Measurement protocol:

• Position: standing upright, arms alongside the body, elbow extended.
• Adjustment: handle adapted to hand size to allow a 90° angle at the proximal interphalangeal joint of the index finger.
• Output: maximal voluntary contraction, reported in kilograms (kg) (Table 5).

#### 2.3.2. Lumbar Strength

Lumbar strength was assessed using the Back D dynamometer (20–300 kg, accuracy 0.5 kg), as an indicator of trunk stability and resistance to mechanical overload. This measurement is particularly important for football players, where lumbar stability prevents excessive spinal loading during sprints, jumps, and contact play.

Measurement protocol:

• Position: standing on the platform with feet shoulder-width apart.
• Initial posture: trunk inclined forward at ~30°, arms extended holding the handle.
• Execution: gradual extension of the trunk while maintaining knees fully extended.
• Output: maximal force (kg) (Table 6).

#### 2.3.3. Spinal Mobility

Spinal flexibility was measured with the Flexion D flexometer (–20 to +35 cm range). The test quantifies the degree of forward flexion of the dorsolumbar spine, reflecting both joint mobility and muscular elasticity.

Measurement protocol:

• Position: standing on a 50 cm-high platform, feet together, arms extended.
• Execution: slow forward flexion without knee bending, pushing the cursor of the flexometer as far as possible.
• Output: maximal displacement (cm) (Table 7).

### 2.4. Biological Evaluation

Biological evaluation provides critical insights into the physiological status of elite youth football players [23]. Hematological, biochemical, and urinary parameters allow monitoring of oxygen transport, metabolic balance, inflammation, and renal adaptation to physical effort [24]. Deviations from reference ranges may indicate overtraining, insufficient recovery, or hidden health risks that can predispose to musculoskeletal injuries [25,26,27].

All analyses were conducted in the morning, in fasting state, using standard clinical laboratory methods. Blood samples were collected via venipuncture, while urine was obtained from first morning void. Parameters were compared with established medical reference ranges and interpreted in the context of sports performance and injury prevention.

#### 2.4.1. Hematological and Biochemical Parameters

The hematological and biochemical profile included indicators of oxygen transport, metabolic function, inflammatory status, and mineral balance (Table 8).

#### 2.4.2. Urinalysis Parameters

Urinalysis provided complementary information on hydration status, renal adaptation, and muscle microtrauma (Table 9).

### 2.5. Statistical and Machine Learning Methods

The analyses were grouped according to the three main domains of data collection: anthropometric, physical, and biological. Within each domain, both classical statistical techniques and advanced multivariate/ML approaches were applied (Table 10). Normality of variables was assessed using the Shapiro–Wilk test and Q–Q plots, while homogeneity of variances was evaluated using Levene’s test. Non-normal variables were log-transformed where necessary to meet parametric assumptions. PCA was applied to the 12 anthropometric variables to reduce dimensionality and identify latent structural components. The first two principal components, explaining 67% of the variance, were retained for interpretation. Factor Analysis was applied to the biological variables to identify metabolic–inflammatory latent domains.

For supervised modeling:

• Linear regression was selected as an interpretable baseline model suitable for medium-sized datasets and continuous outcomes. SVR (RBF kernel) was included to explore potential non-linear relationships not captured by linear models.
• Model performance was evaluated using 5-fold cross-validation rather than a fixed train-test split, improving robustness and reducing overfitting risks.

For unsupervised learning:

• K-means clustering was used to identify hidden adaptation profiles. The optimal number of clusters (k = 3) was determined through the elbow method and silhouette scores, indicating best separation and compactness at k = 3.

An integrative multiblock correlation analysis linked anthropometric PCA components with biological factor scores to explore cross-domain interactions.

All statistical analyses and ML simulations were executed in reproducible notebooks on Kaggle, a managed cloud platform that allows writing and running code end-to-end without any local setup or special environment. The standard scientific Python 3.12 stack (NumPy, pandas, scikit-learn, statsmodels, matplotlib) was used within Kaggle notebooks, ensuring consistent dependencies and easy reruns across sessions.

**Figure 1:**
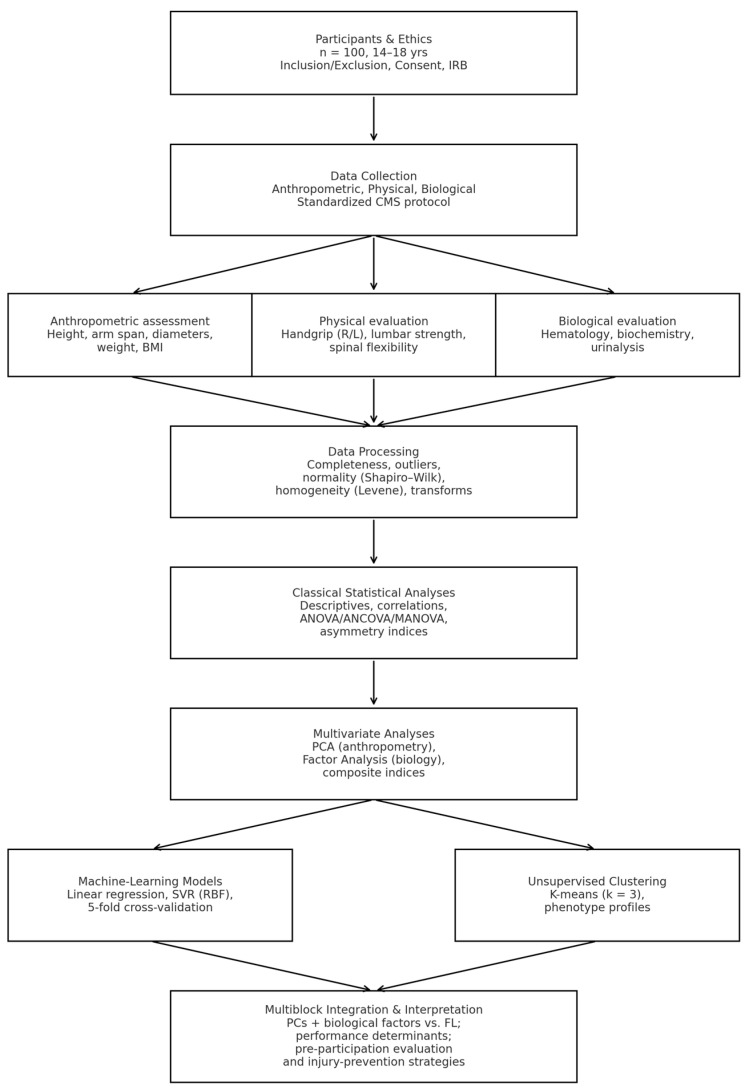
Flowchart of the integrated anthropometric–physical–biological assessment and machine-learning analysis workflow.

The overall workflow of data collection, preprocessing, statistical analysis, and ML modeling is summarized in Figure 1.

## 3. Results

### 3.1. Anthropometric Results

The anthropometric evaluation of the 100 elite youth football players revealed relatively homogeneous body composition parameters, with some inter-lot differences. The overall mean age of the sample was 17.3 ± 2.0 years (range 16–27). Distribution analysis indicated a pronounced right skew (skewness = 2.65, kurtosis = 7.79), with five outliers corresponding to older athletes (>20 years). When stratified by lot, Lot 1 exhibited a significantly higher age profile (19.5 ± 2.9 years) compared with Lot 2 (16.5 ± 0.6 years) and Lot 3 (16.5 ± 0.8 years), confirming that Lot 1 included more mature players.

Body height showed a mean of 178.6 ± 6.6 cm (range 155.5–195.0), with a parallel mean arm span of 179.2 ± 6.7 cm, indicating proportional growth. Both parameters followed a near-normal distribution with minimal skewness. Lot 1 players were taller (182.3 cm) than Lot 2 (178.0 cm) and Lot 3 (176.8 cm), suggesting selection bias toward taller players in Lot 1.

Mean body weight and BMI were 66–70 kg and 21.7 ± 2.2 kg/m^2^, respectively. At the lot level, Lot 1 showed higher BMI values (22.8 kg/m^2^) compared to Lot 2 (20.9 kg/m^2^) and Lot 3 (21.4 kg/m^2^), consistent with their older age and advanced physical development. Other anthropometric indicators (shoulder, thoracic, and chest diameters) displayed low coefficients of variation (4–7%), suggesting homogeneous distributions across the cohort, with only a small number of outliers.

Overall, the descriptive analysis highlighted that Lot 1 comprised taller, heavier, and older athletes with higher BMI, while Lots 2 and 3 were younger and more homogeneous, reflecting differences in biological maturity and selection stage. These findings provide the basis for subsequent correlation and multivariate analyses.

On the whole sample, age was positively associated with multiple anthropometric indicators:

• Anteroposterior and transverse diameters;
• Body weight (r = 0.47, *p* < 0.001) and BMI (r = 0.45, *p* < 0.001), suggesting that older players exhibited more developed somatic features;
• Height and arm span were strongly correlated (r = 0.75, *p* < 0.001), confirming proportional body development, and both were strongly associated with trunk and chest diameters;

At the lot level, different patterns emerged:

• Lot 1 (older group): age correlated significantly with chest diameter (r = 0.46, *p* = 0.019) and BMI (r = 0.57, *p* = 0.003). An arm span–height correlation remained strong (r = 0.73, *p* < 0.001).
• Lot 2 (intermediate group): stronger structural associations were evident, with height correlating with arm span (r = 0.81, *p* < 0.001), shoulder and chest diameters (r > 0.70, *p* < 0.001), and body weight (r = 0.68, *p* < 0.001).
• Lot 3 (younger group): similar but slightly weaker patterns, with arm span–height correlation (r = 0.67, *p* < 0.001) and consistent associations between trunk diameters and body weight (r = 0.43–0.61, *p* < 0.01).

Overall, these results indicate that somatic development is coherent across dimensions, with proportional increases in body mass, height, and skeletal diameters. The correlation of age with body weight and BMI is especially evident in the older subgroup (Lot 1), reflecting biological maturity differences.

#### 3.1.1. Principal Component Analysis (PCA)

**Figure 2:**
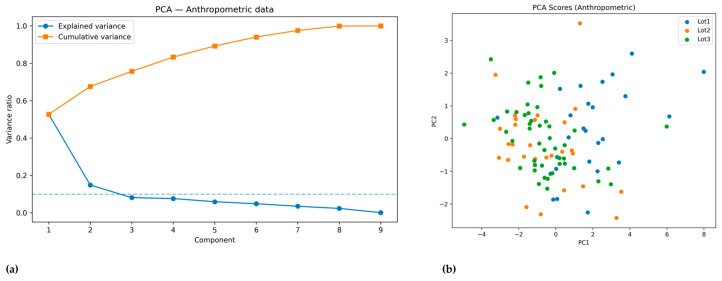
Principal Component Analysis (PCA) of anthropometric variables. (**a**) Scree plot showing the proportion of variance explained by each component; (**b**) PCA score plot illustrating the distribution of players from each lot along the first two principal components.

PCA was performed to reduce dimensionality and identify latent patterns among anthropometric variables. The scree plot (Figure 2a) showed that the first two components explained approximately 67% of the total variance (PC1: 50%, PC2: 17%).

PC1 loaded heavily on height, arm span, weight, BMI, and trunk diameters, representing a general dimension of somatic size and body mass. PC2 was characterized by a contrast between age and body weight (positive loadings) and height/arm span (negative loadings), thus reflecting biological maturity versus proportional growth.

The PCA score plot (Figure 2b) revealed partial group separation. Lot 1 players tended to score higher on PC1 and PC2, consistent with their older age and larger body mass. Lot 2 and Lot 3 clustered more closely, reflecting their younger age and homogeneity in anthropometric development.

Taken together, PCA highlighted that maturity-related body mass and proportional skeletal growth are the main axes of variability in elite youth football players.

#### 3.1.2. MANOVA Between Lots

To evaluate differences in anthropometric profiles across groups, a MANOVA was conducted with Lot (Lot 1, Lot 2, Lot 3) as the independent factor and all anthropometric measures as dependent variables. The results indicated a significant multivariate effect of Lot (Wilks’ λ = 0.497, F(18,178) = 4.13, *p* < 0.001; Pillai’s trace = 0.517, *p* < 0.001; Hotelling–Lawley trace = 0.981, *p* < 0.001; Roy’s greatest root = 0.950, *p* < 0.001).

Follow-up univariate ANOVAs showed significant differences across groups for key measures such as height, weight, BMI, and trunk diameters. Post hoc Tukey HSD comparisons revealed that Lot 1 was significantly taller, heavier, and had higher BMI values compared with both Lot 2 and Lot 3, whereas Lot 2 and Lot 3 differed less markedly, with only modest differences in stature-related measures.

These findings reinforce the interpretation that Lot 1 comprised older, more physically mature athletes, while Lot 2 and Lot 3 represented younger, more homogeneous cohorts.

### 3.2. Physical Results

To evaluate neuromuscular performance and potential asymmetries, we conducted paired t-tests, ANCOVA models with Lot as a fixed factor and age/BMI as covariates, and asymmetry index analyses.

Paired *t*-tests revealed a statistically significant difference between left and right measurements for the ffp variable (t(109) = −4.19, *p* < 0.001). The negative *t*-value indicates that the dominant side performed systematically better than the contralateral limb. This consistent asymmetry suggests a possible imbalance in functional power, which is relevant in the context of injury risk and load management in elite youth football players.

ANCOVA analyses highlighted that the effect of Lot was significant for the fl variable (F(2105) = 12.64, *p* < 0.001). In addition, age emerged as a significant covariate for ffpd, ffps, and fl (all *p* < 0.05), reinforcing the influence of maturational status on physical performance. In contrast, BMI did not show significant effects (*p* > 0.47), suggesting that body composition was less determinant compared to age and developmental stage. Importantly, the influence of Lot remained significant for fl even after adjusting for covariates, indicating genuine intergroup differences.

Asymmetry indices (AIs) calculated for ffp revealed moderate imbalances at the group level. The mean asymmetry index relative to the pair mean was 5.42 ± 5.04%, with median values around 4–5% and a maximum observed asymmetry of 30%. Stratification by Lot indicated similar distributions:

• Lot 1: 5.55 ± 6.29% (median = 4.25%).
• Lot 2: 5.63 ± 5.94% (median = 3.87%).
• Lot 3: 5.21 ± 3.51% (median = 4.57%).

These values are consistent with acceptable asymmetry thresholds (<10%) reported in young athletes. No significant between-lot differences were observed in AI values, confirming that asymmetry was relatively uniform across groups.

Taken together, these findings indicate that (i) a significant functional imbalance exists in lower-limb power (ffp), (ii) intergroup differences in physical performance are primarily explained by lot and age, and (iii) asymmetry remains within acceptable ranges but may still warrant monitoring in load management and injury prevention strategies.

### 3.3. Biological Results

The descriptive analysis of the biological markers revealed overall values within the physiological range, with minor variations between individuals and across groups. Hematological parameters showed a mean hemoglobin concentration of 15.16 g/dL (95% CI: 14.99–15.33) and a hematocrit mean of 43.7% (95% CI: 43.2–44.2), values that are typical for young elite athletes and indicative of a normal oxygen transport capacity. White blood cell counts were also within reference limits (median = 6.38 × 10^9^/L, reference interval: 4.2–10.2 × 10^9^/L), suggesting the absence of underlying infectious or inflammatory conditions in the study cohort.

Biochemical markers further supported a favorable metabolic and physiological profile. Serum glucose values were tightly distributed (mean = 86.7 mg/dL, CI95%: 85.7–87.7), while HDL cholesterol presented higher inter-individual variability (range: 34–74 mg/dL), with a group mean of 52.9 mg/dL. This variability may reflect both genetic predisposition and the impact of training loads on lipid metabolism. Hepatic enzymes presented a moderate spread, with median TGP values of 16 U/L (95% reference interval: 11–31 U/L) and serum transaminase oscillations occasionally exceeding the upper limit (max: 35 U/L), potentially reflecting transient training-induced adaptations.

Markers of inflammation (VSH, CRP) demonstrated a consistently low profile across the entire sample. Median CRP was 0.8 mg/L with an upper reference threshold of 1.4 mg/L, and median VSH was 5 mm/h, both values suggesting a reduced inflammatory state. This is in line with the expected anti-inflammatory adaptations associated with regular high-intensity training in young athletes.

Renal function was preserved, with mean serum creatinine levels of 0.84 mg/dL and mean urea values of 33 mg/dL, well within normal physiological ranges. Electrolyte status was stable, with mean serum calcium at 9.68 mg/dL and magnesium at 1.91 mg/dL, and urine density distributed within narrow physiological limits (mean = 1023 g/L).

The factor analysis (varimax rotation, 6 factors extracted) suggested the presence of several latent domains of biological variability. The strongest loadings indicated three primary clusters: (1) a hematological factor, driven by Hb and Ht; (2) a metabolic/hepatic factor, including liver enzymes (TGP, TO); and (3) a lipidic/renal factor, associated with HDL and urea values. Inflammatory markers (CRP, VSH) did not cluster strongly with the other variables, reflecting their uniformly low distribution. While additional minor factors emerged, their interpretability was limited, likely reflecting noise rather than consistent biological domains.

To facilitate interpretation, composite biological scores were calculated by aggregating standardized z-scores for the main physiological domains (hematology, renal, lipidic, electrolytes, inflammation). These composite indices highlighted clear trends across the cohort. The inflammatory composite consistently yielded negative scores, confirming the low-inflammatory state of the athletes. The lipid composite showed greater variability, with some individuals scoring more than +1 SD above the mean, indicating heterogeneity in lipid metabolism. The hematological composite also varied between subjects, reflecting inter-individual differences in red cell mass and oxygen-carrying capacity, potentially linked to maturational stage or adaptation to training.

In summary, the biological profile of the elite youth football players was characterized by physiological values within normal ranges, with systematically low inflammatory markers, stable renal and electrolyte homeostasis, and moderate variability in lipidic and hepatic parameters. The latent structures extracted from factor analysis and the composite biological indices support the interpretation that young football players display a globally favorable health profile but with inter-individual differences in metabolic and hematological domains that may be relevant for performance monitoring and individualized injury prevention strategies.

### 3.4. Machine Learning Analysis

The integrated ML analysis on the full dataset (anthropometric, physiological, and biological variables) used FL (physical performance index) as the prediction target. Four complementary approaches were implemented: regression-based prediction, kernel methods, unsupervised clustering, and multiblock correlation analyses, followed by cross-validation.

#### 3.4.1. Linear Regression

Linear regression with 5-fold cross-validation provided moderate predictive ability, with a mean R^2^ = 0.59 ± 0.11 and a mean absolute error (MAE) of 10.0 ± 0.8. Performance across folds varied between R^2^ = 0.45 and 0.75, suggesting that approximately 60% of the variance in performance could be explained by the multivariate predictors.

**Figure 3:**
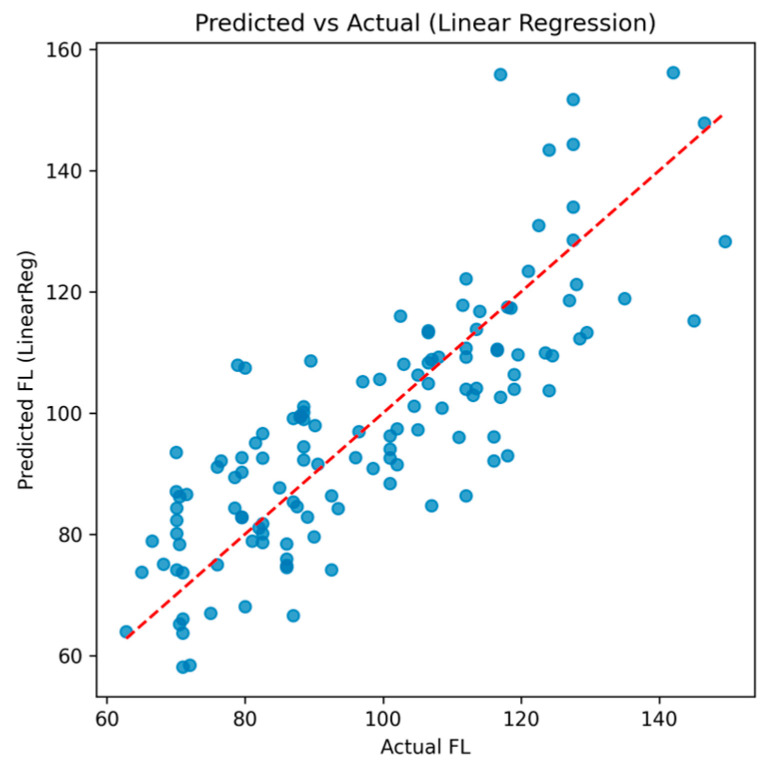
Predicted vs. actual FL values using linear regression.

The scatterplot of predicted versus actual FL values (Figure 3) shows a close alignment with the identity line, indicating stable predictive accuracy.

#### 3.4.2. Support Vector Regression (SVR)

**Figure 4:**
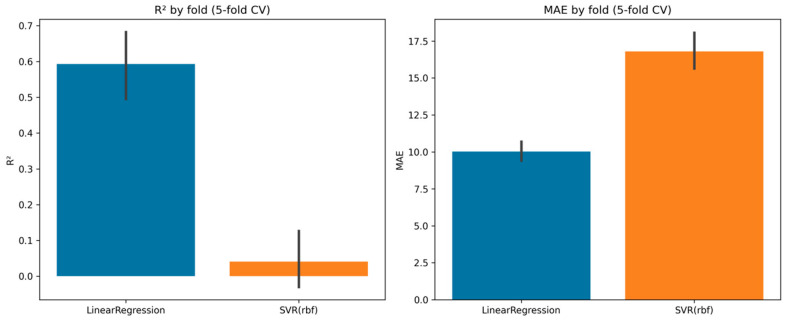
Cross-validated scores (R^2^ and MAE) for linear regression vs. SVR.

By contrast, support vector regression (SVR, RBF kernel) underperformed, with an average R^2^ close to zero (0.04 ± 0.09) and higher error rates (MAE = 16.8 ± 1.4). This indicates that, in this dataset, linear models captured the variance structure more effectively than non-linear kernel-based methods. Model comparison is summarized in Figure 4, which highlights the superiority of linear regression over SVR both in terms of R^2^ and error metrics.

The variability and occasionally negative R^2^ values observed for SVR across folds suggest a tendency towards overfitting and instability in this relatively small sample, especially when using a non-linear kernel. This finding further supports the use of simpler, interpretable linear models for performance prediction in this context.

#### 3.4.3. Unsupervised Clustering

Unsupervised clustering (k-means, k = 3) revealed three distinct subgroups of athletes:

• Cluster 0 (*n* = 67, ~52% of cohort).
• Cluster 1 (*n* = 21, ~16%).
• Cluster 2 (*n* = 42, ~32%).

**Figure 5:**
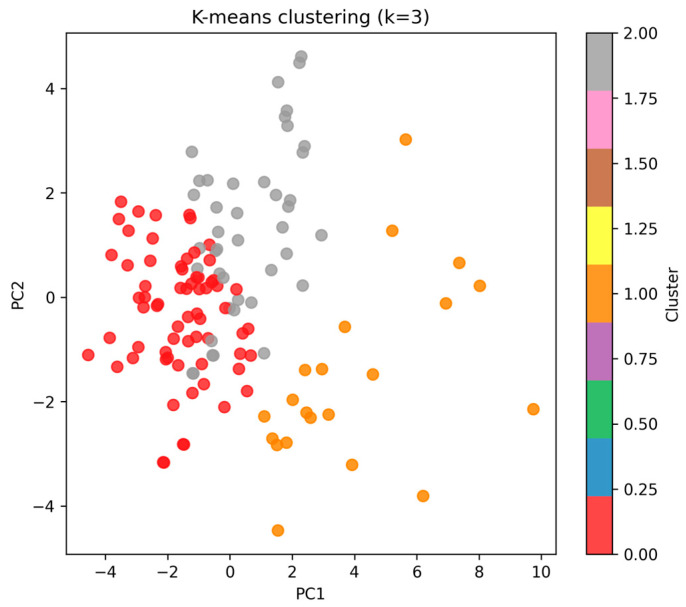
K-means clustering (k = 3) plotted on the first two PCA components.

The projection of the clusters on the first two PCA dimensions (Figure 5) suggests that these subgroups may reflect heterogeneous adaptation profiles, potentially related to training status, biological maturation, or injury risk.

From a practical standpoint, these clusters can support individualized decision making. For example, players in a cluster characterized by larger body size but less favorable biological profiles could benefit from targeted conditioning and recovery interventions, whereas players in clusters with more balanced structural and biological profiles may tolerate standard training loads. Thus, cluster-based profiling offers an intuitive tool for technical and medical staff when planning training load and monitoring adaptation.

#### 3.4.4. Multiblock Correlation Analysis

**Figure 6:**
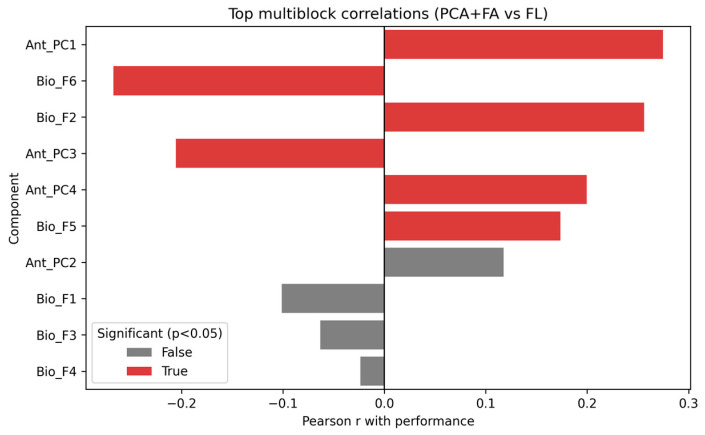
Multiblock correlations between PCA (anthropometry), FA (biology) scores, and FL.

Integration of anthropometric PCA scores and biological factor analysis (FA) scores revealed several significant associations with performance (Figure 6). The strongest predictors were:

• Ant_PC1 (r = 0.28, *p* = 0.002), reflecting general anthropometric variance.
• Bio_F6 (r = −0.27, *p* = 0.002) and Bio_F2 (r = 0.26, *p* = 0.003), capturing relevant biological domains.
• Ant_PC3 (r = −0.21, *p* = 0.019) and Ant_PC4 (r = 0.20, *p* = 0.023).

These results highlight that both anthropometric and biological latent factors contribute significantly to physical performance prediction. Specifically, the combination of anthropometric variability (PC1, PC3, PC4) and specific biological composites (lipid-metabolic and renal factors) appears to explain performance differences.

The 5-fold validation confirmed the robustness of the regression model, with relatively stable R^2^ and MAE across folds. SVR performance, in contrast, was unstable and consistently inferior (Table 11).

In summary, the ML approach confirmed that linear models outperform non-linear kernels for performance prediction in this cohort, explaining approximately 60% of the variance while maintaining stability across validation folds.

## 4. Discussion

### 4.1. Key Findings and Integrated Interpretation

This study offers an integrated portrait of anthropometric, physical, and biological characteristics in elite youth football players (*n* = 100, 14–18 yrs), enhanced with AI/ML analytics. Anthropometric variability was organized along two dominant axes: a size–mass dimension (stature/arm span/body mass/trunk diameters) and a maturity–proportionality contrast, which differentiated lots—Lot 1 appearing older and larger. Physically, we identified a systematic right–left difference for the ffp metric (mean AI ≈ 5%), and lot-related differences in the composite performance index (fl) that persisted after adjusting for age and BMI, with age emerging as a key covariate. Biologically, markers were within reference ranges, with low inflammatory tone (CRP, ESR), stable renal–electrolyte status, and moderate dispersion in lipidic/hepatic indicators. Factor analysis suggested interpretable latent domains (hematologic; hepatic–metabolic; lipid/renal), while inflammation contributed little to variance—consistent with the low-inflammatory state observed [28].

These patterns align with previous work showing that biological maturation is a key determinant of anthropometric proportionality and neuromuscular performance in youth football players, and that elite academies tend to select taller and more physically mature athletes for older age groups. Similarly, the asymmetry levels observed in our cohort (<10% at the group level, with a small number of higher outliers) are comparable to those reported in studies of adolescent and young adult players, where moderate asymmetry is considered physiologically acceptable but may still warrant monitoring in individuals with extreme values.

In prediction tasks, linear regression achieved 5-fold R^2^ = 0.59 ± 0.11 (MAE = 10.0 ± 0.8), outperforming SVR (RBF), indicating that dominant relationships are approximately linear and well-captured after latent-space engineering (PCA/FA). K-means (k = 3) revealed three phenotypes that plausibly reflect training history/maturity/coping with load. Multiblock correlations confirmed that both anthropometric PCs (PC1, PC3/4) and biological factors (Bio_F2/F6) relate to performance, endorsing a systems perspective: performance variance emerges from cross-domain coupling, not from any single silo.

The superiority of linear over non-linear models is consistent with recent ML applications in biomedical and sports datasets of similar size, where simpler algorithms often yield more stable and interpretable predictions than complex kernel-based methods. Our findings support the idea that, once latent anthropometric and biological components are extracted, the remaining relationships with performance can largely be modeled in a linear space.

### 4.2. Practical Implications for Performance and Injury Prevention

Findings support a three-layered prevention strategy readily adoptable in academies:

• Structure (Anthropometry): track proportionality indices (e.g., arm span–height; shoulder–pelvis balance) and use maturity-adjusted references to avoid conflating growth with performance.
• Function (Physical): monitor symmetry longitudinally; although group AI is acceptable (~5%), individual outliers (up to ~30%) warrant corrective microcycles (unilateral strength, motor control, landing mechanics). Lot and age effects on fl justify age-/maturity-aware benchmarking.
• State (Biology): use composite indices (hematology, hepatic–metabolic, lipid/renal, electrolytes, inflammation) as a concise dashboard to tailor nutrition, hydration, and recovery—especially when lipid/renal composites deviate (e.g., >+1 SD) under rising loads.

Because the linear model is both accurate and interpretable, it facilitates transparent staff communication (e.g., thresholds on PCs/factors) and actionable decision rules (green/amber/red flags). The cluster phenotypes can guide individualized programming and tracking of intervention response.

From an applied perspective, the three k-means clusters provide an intuitive framework for tailoring intervention. For example, a cluster characterized by larger body size and less favorable lipid/renal profiles may be prioritized for individualized conditioning, nutritional counseling, and tighter load management, whereas clusters with balanced structural and biological scores may be managed with standard training plans. This type of profiling can be incorporated into pre-participation evaluations and periodic reviews, helping coaches and medical staff to align training loads, recovery strategies, and medical follow-up with each player’s adaptation profile.

### 4.3. Strengths, Limitations, and Future Directions

Strengths include tri-domain integration under one standardized protocol, latent-space methods (PCA/FA) paired with interpretable prediction and robust 5-fold validation, and the operationalization of biological composites plus unsupervised phenotypes for applied decision making.

Limitations: The cross-sectional design limits causal inference; the fl outcome aggregates multiple capacities (task-specific targets could refine granularity); SVR underperformance may reflect sample size and hyperparameter constraints (future work: Elastic Net, nested CV, broader kernel search). External validity is currently limited to male adolescent academies of similar profiles.

In addition, the sample size, although adequate for PCA, factor analysis, and linear regression, may not be sufficient to fully exploit high-capacity non-linear models without overfitting. Training load, wellness, and contextual variables (e.g., match congestion, positional role) were not systematically included in the present analysis, which may partly explain the residual unexplained variance in performance. Finally, injury outcomes were not prospectively tracked, so the identified phenotypes and predictors cannot yet be directly interpreted as injury-risk markers.

Future work should implement prospective, season-long monitoring to test whether composites/PCs and the linear model predict injuries and availability [29]. Adding training load (GPS, session-RPE) and sleep/recovery metrics should enhance prediction [30]. Transitioning from k-means to supervised risk models (e.g., survival analysis for time-to-injury; ordinal risk bands) and deploying a lightweight, explainable decision-support tool could translate this framework into daily high-performance operations [31].

Longitudinal designs should specifically examine how fluctuations in biological markers (e.g., CRP, ESR, lipid and renal indices) interact with external and internal load to anticipate periods of maladaptation, and whether threshold-based or trajectory-based ML models can predict time-loss injuries or reduced availability. Integrating routine pre-participation screening with continuous monitoring across a full competitive season will be crucial for validating the practical utility of the present composite indices, latent factors, and linear prediction models.

### 4.4. Overall Summary

In adolescent elite football, maturity-adjusted anthropometry, functional symmetry, and balanced biological domains jointly shape performance and resilience [32]. Simple, validated linear models, fueled by latent factors from anthropometry and biology, deliver accurate and interpretable predictions, while unsupervised clustering exposes meaningful phenotypes for individualized care. Embedding these elements into routine screening and load management may help reduce injury risk and optimize development pathways during the critical youth window.

Overall, our findings support the integration of multidimensional profiling and interpretable ML tools into standard pre-participation evaluation and periodic medical screening in elite youth academies. Rather than replacing clinical judgment, such tools can complement the expertise of medical and technical staff by highlighting players who deviate from expected structural, functional, or biological patterns and by providing an objective basis for individualized prevention strategies.

## 5. Conclusions

This study demonstrates that an integrated evaluation of anthropometric, physical, and biological parameters, enhanced by ML, provides a comprehensive framework for monitoring elite youth football players.

Anthropometric analysis revealed consistent patterns of proportional growth and maturity-related differences, while physical testing highlighted systematic asymmetries and age-related performance effects. Biological profiling confirmed globally favorable health and recovery status, with low inflammatory markers and moderate variability in metabolic and lipid domains.

ML analyses showed that linear regression explained ~60% of the variance in performance, outperforming more complex non-linear models, and that multiblock correlations confirmed the complementary contribution of both structural and biological domains. K-means clustering further identified distinct adaptation phenotypes, underlining the heterogeneity within the cohort.

Taken together, these findings support the inclusion of combined anthropometric, functional, and biological markers in routine pre-participation evaluation, where they can enhance early risk detection, individualize training loads, and improve decision making in youth football academies.

Strengths of this work include the multidimensional design, the use of latent-space methods, and the application of interpretable ML models with robust cross-validation. However, limitations must be acknowledged: the cross-sectional design prevents causal inference; performance was summarized in a composite index; and the dataset size limits the reliability of more complex ML algorithms.

Future research should incorporate longitudinal monitoring across full competitive seasons to test the predictive value of latent factors and biological composites for injuries and player availability, as well as to integrate external load (GPS), internal load, and recovery metrics into holistic predictive models.

In summary, this study provides a practical, interpretable, and multidimensional framework that can guide personalized monitoring and developmental strategies in adolescent football players, helping medical and technical staff optimize performance and reduce injury risk during critical stages of growth.

## Figures and Tables

**Table 1:** Descriptive statistics of anthropometric, physical, and biological variables (*n* = 100).

Variable	Mean	SD	Min	Max
ANTHROPOMETRIC VARIABLES				
Age (years)	17.28	2.04	16.00	27.00
Height (cm)	178.58	6.65	155.50	195.00
Arm span (cm)	179.22	6.70	164.00	195.00
Biacromial diameter (cm)	41.51	1.87	37.00	47.00
Thoracic transverse diameter (cm)	32.64	2.21	29.00	44.00
Thoracic sagittal diameter (cm)	27.56	2.02	21.00	33.00
Anteroposterior diameter (cm)	19.95	1.53	16.00	25.00
Body mass (kg)	69.12	9.16	49.00	98.00
BMI (kg/m^2^)	21.58	2.30	16.67	27.65
PHYSICAL PERFORMANCE VARIABLES				
FFPD—Dominant limb power (cm)	38.44	6.25	23.10	62.30
FFPS—Non-dominant limb power (cm)	37.49	6.44	22.00	54.60
FL—Performance index	99.46	20.98	62.80	149.50
BIOLOGICAL VARIABLES				
Hemoglobin (g/dL)	15.14	0.86	12.10	16.90
Hematocrit (%)	43.64	2.47	35.80	48.50
Leukocytes (×10^9^/L)	6.79	1.52	4.21	13.60
ALT/TGP (U/L)	17.35	5.52	9.00	35.00
AST/TGO (U/L)	26.01	7.49	15.00	65.00
Fasting glucose (mg/dL)	87.17	5.76	69.00	99.00
HDL cholesterol (mg/dL)	51.75	9.82	32.00	74.00
ESR/VSH (mm/h)	5.59	2.22	3.00	20.00
CRP (mg/L)	0.81	0.62	0.50	4.80
Urea (mg/dL)	33.86	8.43	16.00	53.00
Creatinine (mg/dL)	0.85	0.12	0.60	1.22
Calcium (mg/dL)	9.68	0.39	7.90	10.60
Magnesium (mg/dL)	1.91	0.28	1.00	2.90
Urine specific gravity (g/L)	1023.73	5.00	1006.00	1031.00

**Table 2:** Normal reference values for anthropometric parameters (16–35 years).

Parameter	Women	Men	Relevance
Height (stature)	155–175 cm	165–185 cm	Outliers increase joint stress
Arm span	≈height ± 3–5 cm	≈height ± 3–5 cm	Disproportion → postural risk
Biacromial diameter	36–38 cm	40–44 cm	Shoulder–pelvis balance
Bitrochanteric diameter	28–32 cm	26–30 cm	Hip–knee mechanics
Thoracic transversal diameter	24–28 cm	28–32 cm	Breathing, posture
Thoracic sagittal diameter	16–20 cm	18–22 cm	Postural stability

**Table 3:** Nutritional parameters, measurement protocols, and relevance.

Parameter	Description	Measurement Protocol	Relevance for Football Players/Injury Prevention
Body weight	Total body mass, reflecting muscular and fat tissue	Measured with calibrated scale, in the morning, barefoot, minimal clothing	Overweight ↑ stress on lower limb joints; underweight → insufficient muscle mass and reduced power
BMI	Weight-to-height ratio (kg/m^2^)	Calculated as weight (kg)/height^2^ (m^2^)	High BMI → potential obesity, ↑ injury risk; Low BMI → reduced energy reserves, fatigue

**Table 4:** Reference nutritional values for performance parameters (16–35 years).

Parameter	Normal Range	Notes
Body weight	≈height (cm)–100 ± 10%	Adjusted for sex and somatotype
BMI	18.5–24.9 kg/m^2^	Should be interpreted with caution in athletes due to high muscle mass

**Table 5:** Reference values for nutritional parameters (16–35 years).

Sex	Normal Range (kg)	Relevance for Football Players
Male	40–60	Marker of global strength; low values → reduced stability, fatigue risk
Female	20–35	Same role, lower absolute thresholds

**Table 6:** Lumbar strength—reference values (16–35 years).

Sex	Normal Range (kg)	Relevance for Football Players
Male	120–180	Essential for trunk stabilization; low values → predisposition to low back pain
Female	70–120	Lower absolute force, but relative to body weight, is critical for injury prevention

**Table 7:** Spinal mobility—reference values (16–35 years).

Sex	Normal Range (cm)	Relevance for Football Players
Male	9–13	Hypomobility → rigidity, higher risk of contractures; hypermobility → instability, entorses
Female	13–15	Better flexibility; extreme values must be interpreted cautiously

**Table 8:** Hematological and biochemical parameters, description, and relevance.

Parameter	Physiological Role	Relevance for Football Players/Injury Prevention	Reference Values (16–35 yrs)
Hemoglobin (Hb)	Oxygen transport	Low values → fatigue, ↓ aerobic capacity, ↑ muscle injury risk	M: 13–18 g/dL; F: 12–16 g/dL
Hematocrit (Ht)	Proportion of red cells	Low → hypoxia; High → blood viscosity ↑ thrombosis risk	M: 40–52%; F: 36–46%
Leukocytes (WBC)	Immune response	Elevated → infection/inflammation; ↓ recovery delays	4000–10,000/µL
ALT (TGP), AST (TGO)	Hepatic and muscle enzymes	↑ values → muscle microdamage or liver overload	<40 U/L
Fasting glucose	Energy supply	Low → hypoglycemia, cramps; High → poor metabolic control	70–100 mg/dL
HDL cholesterol	Cardiovascular health	Low → poor oxygenation and recovery	M: >40 mg/dL; F: >50 mg/dL
LDL cholesterol	Lipid transport	High → cardiovascular stress	<130 mg/dL (optimal <100)
ESR (VSH)	Chronic inflammation	↑ → slow tissue recovery, tendinopathies	M: 2–15 mm/h; F: 2–20 mm/h
CRP	Acute inflammation	↑ → muscle microtrauma, overload	<5 mg/L
Urea	Protein metabolism	↑ → catabolism, muscle fatigue	15–45 mg/dL
Creatinine	Renal function, muscle mass	↑ → dehydration, muscle overload	M: 0.6–1.3 mg/dL; F: 0.5–1.1 mg/dL
Calcium	Muscle contraction, bone health	Deficit → cramps, fractures	8.5–10.2 mg/dL
Magnesium	Neuromuscular relaxation	Deficit → spasms, cramps	1.6–2.6 mg/dL

**Table 9:** Urinalysis parameters, description, and relevance.

Parameter	Description	Measurement Protocol	Relevance for Football Players/Injury Prevention
Proteinuria	Marker of renal/muscle integrity	Presence → microtrauma or renal stress	Absent/<150 mg/24 h
Hematuria	Red blood cells in urine	Indicates trauma, dehydration, or extreme effort	Absent/0–2 RBC per field
Specific gravity	Hydration balance	High → dehydration; Low → overhydration	1.005–1.030

**Table 10:** Summary of statistical and ML methods applied to data domain.

Domain	Classical Statistics	Multivariate/ML Methods	Aim of Analysis
Anthropometric	Descriptive statistics; Pearson correlations; ANOVA/ANCOVA (lot comparison, covariates: age, BMI); MANOVA (multivariate differences)	PCA (dimensionality reduction); Multiblock correlations (PCA scores vs. performance); K-means clustering (body proportionality profiles)	Characterize body proportions, test group differences, identify latent dimensions, link structure with performance.
Physical	Paired t-test (right–left comparison); ANOVA/ANCOVA (lot comparison, covariates: age, BMI); Asymmetry indices	Regression models (linear, SVR); K-fold cross-validation; K-means clustering	Evaluate muscular strength and flexibility, detect asymmetries, model performance predictors, and validate models.
Biological	Descriptive statistics with reference intervals; ANOVA/ANCOVA (lot comparison, covariates: age, BMI)	Factor Analysis (latent domains); Composite indices (inflammation, lipid, renal, hematology); Multiblock correlations (FA factors vs. performance)	Assess physiological adaptation, extract latent biological domains, build integrative indices, and link them to performance.

**Table 11:** 5-fold cross-validation results for linear regression and SVR.

Fold	R^2^ (LinearReg)	MAE (LinearReg)	R^2^ (SVR, rbf)	MAE (SVR, rbf)
1	0.753	9.69	0.176	17.21
2	0.699	9.81	−0.037	19.25
3	0.553	9.00	0.105	15.38
4	0.509	10.31	−0.001	15.38
5	0.452	11.28	−0.038	16.72
Mean ± SD	0.593 ± 0.114	10.02 ± 0.75	0.041 ± 0.085	16.79 ± 1.43

## Data Availability

The datasets presented in this article are not readily available because they contain sensitive health information from minors and are subject to GDPR and institutional ethics restrictions. Requests to access the datasets should be directed to the corresponding author.

## Abbreviations

The following abbreviations are used in this manuscript:

AI	Asymmetry Index
ALT	Alanine Aminotransferase
ANCOVA	Analysis of Covariance
ANOVA	Analysis of Variance
Ant_PCk	Anthropometric Principal Component k
AST	Aspartate Aminotransferase
Bio_Fk	Biological Factor k
BMI	Body Mass Index
Ca	Calcium
CI	Confidence Interval
CMS	Standard protocol within this study
CRP	C-Reactive Protein
Cr	Creatinine
CV	Cross-Validation
ESR	Erythrocyte Sedimentation Rate
FA	Factor Analysis
FFP	(Lower-limb) Functional Power
FFPD/FFPS	FFP Dominant/FFP Non-dominant
FL	(Composite) Physical Performance Index
GPS	Global Positioning System
Hb	Hemoglobin
HDL	High-Density Lipoprotein Cholesterol
HemU	Hematuria
Ht	Hematocrit
ISAK	International Society for the Advancement of Kinanthropometry
LDL	Low-Density Lipoprotein Cholesterol
MAE	Mean Absolute Error
MANOVA	Multivariate Analysis of Variance
Mg	Magnesium
ML	Machine Learning
PCA	Principal Component Analysis
ProtU	Proteinuria
R^2^	Coefficient of Determination
RBF	Radial Basis Function
RPE	Rating of Perceived Exertion
SD	Standard Deviation
SVR	Support Vector Regression
USG	Urine Specific Gravity
WBCs	White Blood Cells
